# Dual-YOLO Architecture from Infrared and Visible Images for Object Detection

**DOI:** 10.3390/s23062934

**Published:** 2023-03-08

**Authors:** Chun Bao, Jie Cao, Qun Hao, Yang Cheng, Yaqian Ning, Tianhua Zhao

**Affiliations:** 1 Bionic Robot Key Laboratory of Ministry of Education, School of Optics and Photonics, Beijing Institute of Technology, Beijing 100081, China; 2 Yangtze Delta Region Academy, Beijing Institute of Technology, Jiaxing 314003, China; 3 School of Opto-Electronic Engineering, Changchun University of Science and Technology, Changchun 130022, China

**Keywords:** infrared object detection, dual-YOLO, attention fusion, fusion shuffle, fusion loss

## Abstract

With the development of infrared detection technology and the improvement of military remote sensing needs, infrared object detection networks with low false alarms and high detection accuracy have been a research focus. However, due to the lack of texture information, the false detection rate of infrared object detection is high, resulting in reduced object detection accuracy. To solve these problems, we propose an infrared object detection network named Dual-YOLO, which integrates visible image features. To ensure the speed of model detection, we choose the You Only Look Once v7 (YOLOv7) as the basic framework and design the infrared and visible images dual feature extraction channels. In addition, we develop attention fusion and fusion shuffle modules to reduce the detection error caused by redundant fusion feature information. Moreover, we introduce the Inception and SE modules to enhance the complementary characteristics of infrared and visible images. Furthermore, we design the fusion loss function to make the network converge fast during training. The experimental results show that the proposed Dual-YOLO network reaches 71.8% mean Average Precision (mAP) in the DroneVehicle remote sensing dataset and 73.2% mAP in the KAIST pedestrian dataset. The detection accuracy reaches 84.5% in the FLIR dataset. The proposed architecture is expected to be applied in the fields of military reconnaissance, unmanned driving, and public safety.

## 1. Introduction

In recent years, infrared detection technology has been widely applied in military, remote sensing, civil, and other fields, such as infrared reconnaissance and early warning, infrared space detection, automotive navigation, medical infrared detection, and many other application scenarios. As a critical technology in the field of infrared early warning detection, infrared object detection algorithms adapted to different complex scenes have been widely studied by researchers. Under the situation that the spatial resolution of the optical system is complex to further improve, it is of great significance to study the infrared object detection algorithm with a low false alarm rate and strong adaptability, which is suitable for different scenes.

However, the detection of infrared images also has many challenges. First, the object has fewer features available. Secondly, the signal-to-noise ratio of the image is low. Finally, the real-time performance of infrared image object detection is limited. These factors indicate that designing an object detection network with high accuracy and good real-time performance in infrared images is challenging. We can see from the current research interests that the most popular object detection methods mainly focus on visible scenes, such as Single Shot Detection (SSD) [1], You Only Look Once (YOLO) series [2,3], Fully Convolutional One-Stage (FCOS) Object Detection [4], and other single-stage object detection networks. Furthermore, two-stage object detection algorithms such as Faster R-CNN [5] and Task-aligned One-stage Object Detection (TOOD) [6] exist. In addition, there are also some object detection methods established on anchor-free [7] or transformer [8]. These methods perform well on visible images, but there are always limitations for infrared image detection.

Although there are challenges for infrared target detection, many methods have been tried, and these methods have achieved relatively good results. For example, the YOLO-FIRI [9] algorithm, by improving the YOLOv5 [10] practice, proposed a region-free infrared image object detection method and reached the advanced level on the KAIST [11] and FLIR [12] datasets. The work of I-YOLO [13] is aimed explicitly at infrared object detection on the road. I-YOLO combines DRUNet [14] with YOLOv3 [2] to enhance the infrared image through DRUNet, and finally uses YOLOv3 for accurate object recognition. This method not only has excellent advantages in precision and speed. In the scene of infrared object detection, air-to-ground detection is also a hot issue of single infrared image detection. In [15], Jiang et al. proposed a UAV object detection framework for infrared images and video. The feature is extracted from the ground object, and the improved YOLOv5s is used for object recognition. This infrared recognition method can achieve 88.69% recognition accuracy and 50 FPS speed. The IARet [16] performs well in single infrared image object detection, and the Focus module is designed to improve the detection speed. The IARet is also lightweight, with the entire model measuring just 4.8 MB. For example, many object detection methods are only for a single infrared image. Although they have achieved good results, their common problem is that the single infrared image object detection ability is limited, the feature loss of the object is severe, and the false alarm rate is high.

As we all know, producing visible images requires compensation for external illumination when the illumination conditions are poor. Infrared cameras can produce infrared spectral images throughout the day, but infrared spectral images lack details such as texture and color. Moreover, in infrared images, the critical factor determining the object’s visibility is the temperature difference between the object and the environment. For example, the car object is brighter than the background [17,18]. However, when there are some non-object heat points, it will also lead to the false detection of the object. Therefore, infrared and visible images have advantages and are complementary in information distribution. Combining the unique benefits of visible images with infrared images can compensate for the lack of precision reduction caused by infrared image object detection.

According to the above analysis, some researchers began to try to make complementary detection between infrared and visible images. For example, MFFN [19] proposes a new multi-modal feature fusion network, which uses morphological features, infrared radiation, and motion features to compensate for the deficiency of single-modal detection of small infrared objects. At the same time, MFFN also proposed a characteristic pyramid structure with layer hopping structure (SCFPN) in morphology. In addition, the network’s backbone integrates SCFPN and the voided convolutional attention module into Resblock. This design also gives the network a detection accuracy of 92.01% on the OEDD dataset. However, not all fusion features are helpful. There are also a lot of research works in progress for how to solve the problems caused by fusion features, such as TIRNet [20]. To solve the problem of information redundancy in the fusion of infrared and visible images, RISNet [17] designed a new mutual information minimization module to reduce redundancy. In addition, the RISNet proposed a classification method of light conditions based on histogram statistics. This method automatically classifies more detailed lighting conditions to facilitate the complementary fusion of infrared and RGB images. This design also makes RISNet better than the state-of-the-art methods for infrared image detection, especially under conditions of insufficient illumination, complex background, and low contrast. In addition, the PearlGAN [21] also plays a role in promoting infrared and visible image fusion detection. PearlGAN designed a top-down guided attention module to make the corresponding attention loss reach the hierarchical attention distribution, reduce local semantic ambiguity, and use context information for image coding. Moreover, PearlGAN introduces a structured gradient alignment loss. This design has a good performance effect in the image translation task and provides a new idea for infrared object detection. Like PearlGAN’s constraint design on the loss function of infrared and visible image fusion detection, there are many excellent works, such as CMPD [22].

We propose the Dual-YOLO method based on the above observations on visible image object detection and infrared and visible image fusion detection. This method effectively solves the problems of low accuracy, feature loss, too many fused redundant features, and slow detection speed in infrared image object detection. Compared with the general target detection, our proposed Dual-YOLO is more suitable to solve the problem of target detection based on RGB UAV imagery. We can also see from [23] that target detection based on RGB UAV imagery is more challenging than general target detection. For targets with complex backgrounds, dense distribution, and small size, such as crop quality detection, the detection method based on RGB UAV imagery can improve the detection accuracy. In summary, the main contributions of this paper are listed as follows:

(1) Based on the current YOLOv7 [3] network with the highest accuracy in real-time object detection, we propose the dual-branch that includes an infrared and visible object detection network named Dual-YOLO. This method alleviates the problem of missing texture features in object detection of a single infrared image. The detection accuracy is improved by complementing the infrared and visible image feature information.

(2) We propose the attention fusion module, which added the Inception module and SE mutual attention module in the infrared and visible feature fusion process. So that infrared and visible images can achieve the best feature complementarity and fusion effect without increasing the number of parameters.

(3) We propose the fusion shuffle module, which adds dilated convolution in the infrared and visible feature fusion process and increases the receptive field for feature extraction of the fusion module. In addition, we add the channel shuffle module to make the infrared and visible features more uniform and reduce redundant features. In addition, we design a feature fusion loss function to accelerate the convergence of Dual-YOLO.

(4) Our method achieves state-of-the-art results on the challenging KAIST multispectral pedestrian dataset and the DroneVehicle [24] remote sensing dataset. Moreover, experiments on a multispectral object detection dataset FLIR also demonstrate the effectiveness and versatility of our algorithm.

The rest of this paper is structured as follows: In Section 2, we describe the network structure and methods in detail. Section 3 gives the details of our work and experimental results and related comparison to verify the effectiveness of our method. Finally, we summarize the research content in Section 4.

## 2. Methods

### 2.1. Overall Network Architecture

**Figure 1:**
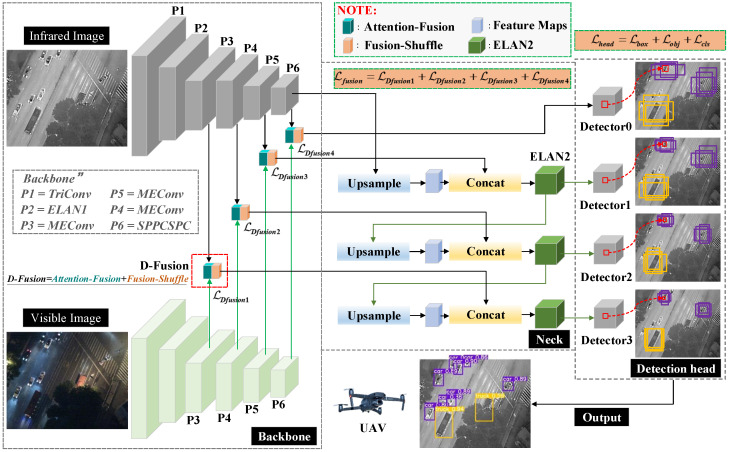
The overall architecture of the proposed Dual-YOLO. The proposed network is mainly designed to detect weak infrared objects captured by UAVs. However, to compensate for the loss of features due to variations in light intensity, we add a visible image feature extraction branch to the network to reduce the probability of missing objects.

**Figure 2:**
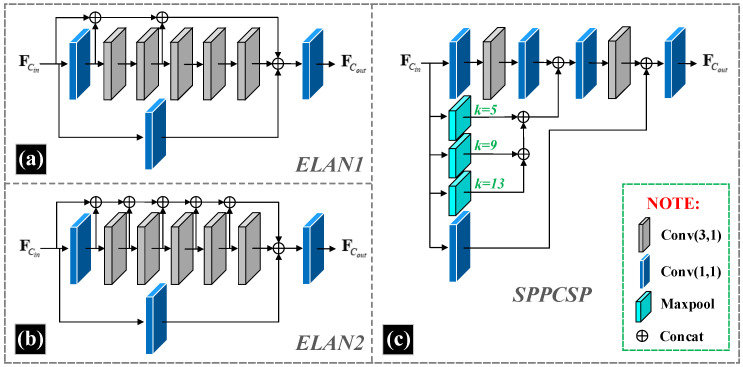
Structures of the feature extraction modules in the backbone and neck. Where (**a**) is the structure of ELAN1, (**b**) is the structure of ELAN2, and (**c**) is the structure of SPPCSP. These structures remain consistent with the design in YOLOv7, where ELAN2 has essentially the same essential components as ELAN1, but ELAN2 has more channels than ELAN1 in the feature aggregation part to ensure that multi-scale feature information is aggregated in the neck. For the maxpool structure in SPPCSP, the value of k is the ratio of downsampling.

The overall network structure we have designed is shown in Figure 1. For the base structure, we take reference from the design of YOLOv7. In the backbone of the object detection network Dual-YOLO, we use P1 to P6 for hierarchical identification. Where the P1 layer uses the TriConv structure. TriConv consists of a three-layer convolution structure with the following format as shown in Equation (1). Where $F_{C_{i}}\in R^{C_{in}\times H_{in}\times W_{in}}$. $F_{C_{i}}$ are the input feature maps, $Conv_{3\times 3}$ representing a convolution operation with kernel size of 3 × 3 and stride 1, and $Conv_{3\times 2}$ representing a convolution operation with kernel size of 3 × 3 and stride 2. The P2 layer uses the ELAN1 structure of YOLOv7, as shown in Figure 2a. The P3 layer uses a combination of MPConv and ELAN1, which we have identified as MEConv. MEConv is calculated as shown in Equation (2). Where the composition of MPConv is shown in Equation (3) and $Conv_{1\times 1}$ representing a convolution operation with kernel size of 1 × 1 and stride 1. The design of the P6 layer is derived from the SPPCSPC structure of YOLOv7 is shown in Figure 2c.

(1) $$TriConv\left(F_{C_{i}}\right)=Conv_{3\times 3}\left(Conv_{3\times 3}\left(Conv_{3\times 3}\left(F_{C_{i}}\right)\right)\right)$$

(2) $$MEConv\left(F_{C_{i}}\right)=MPConv\left(ELAN1\left(F_{C_{i}}\right)\right)$$

(3) $$MPConv\left(F_{C_{i}}\right)=Concat(Conv_{1\times 1}\left(Maxpool\left(F_{C_{i}}\right)\right),Conv_{3\times 2}\left(Conv_{1\times 1}\left(F_{C_{i}}\right)\right))$$

Thermal infrared images have strong feature contrast properties in environments with low light levels. However, visible images have a unique texture feature under normal light conditions. This textural feature can compensate for the lack of recognition of objects in thermal infrared images. Therefore, we add a visible feature extraction branch to the backbone design. The structure of the visible feature extraction branch is the same as that of the infrared feature extraction branch. In the neck’s design, we elicit feature map vectors from the backbone’s P3, P4, P5, and P6. The structure of this type of FPN already covers small, medium, and large objects in the infrared image, thus reducing the probability of missing detection. We design the novel Dual-Fusion (D-Fusion) module in the fusion features section, where the structure and characteristics of D-Fusion are described amply in Section 2.2. The D-Fusion module consists of two parts, Attention-Fusion and Fusion-Shuffle. Furthermore, the Attention-Fusion module is designed to weigh the visible features as the attention feature vector under the attention mechanism with the infrared features. The inspiration for creating the attention fusion module came from our preliminary experiments, where we found a significant miss-detection rate when training and detecting visible or infrared images alone.

In the design of the neck section, we refer to the structure of YOLOv7. Three up-sampling operations are performed in the deck to eliminate the problem of gradual loss of features due to convolution. At the same time, four detection heads are designed to preserve the small object features in the convolution, where the detection head can cover all object sizes.

### 2.2. Information Fusion Module

**Figure 3:**
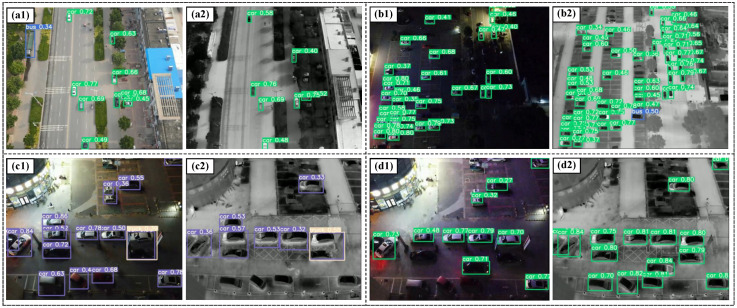
The effect of separate detection of infrared images and visible images. **a(1)**, **a(2)**, **c(1)**, and **c(2)** are training and detection results for single visible data. **b(1)**, **b(2)**, **d(1)**, and **d(2)** are training and detection results for single infrared data. This is a collection of images taken from a drone. The images are taken during the day and night. The drone flies at altitudes of 100 m and 200 m.

The design of this module is derived from several sets of experiments we have conducted on the effectiveness of network detection for a single data source. Before designing Dual-YOLO, we complete the following groups of experiments, as shown in Figure 3. For the single visible image data training model, as in Figure 3a(1), the bus class (blue box) is detected in daylight conditions, and the car class is near the bush. In Figure 3a(2), however, classes such as cars are missed. Furthermore, compared to Figure 3c(1) and c(2), after training the model with single visible images at night when there is not enough light, most objects can be detected, although there are missed detections. However, for infrared images, there are many missed and faulty objects. For the training of infrared images, as in Figure 3b(1), the objects in the car category are submerged in the background due to the faint brightness of the overall image. This phenomenon also leads to a large number of objects being missed. In contrast, in Figure 3b(2), the object of the car class differs significantly from the background features in the thermal infrared image. Therefore, the network has a strong recognition ability when trained with infrared images. Similarly, objects are detected in Figure 3d(2) that are not detected in the visible image case in Figure 3d(1). As a result, the ideal model we want to design is characterized by solid robustness and a meager leakage rate at different light intensities.

#### 2.2.1. Attention Fusion Module

**Figure 4:**
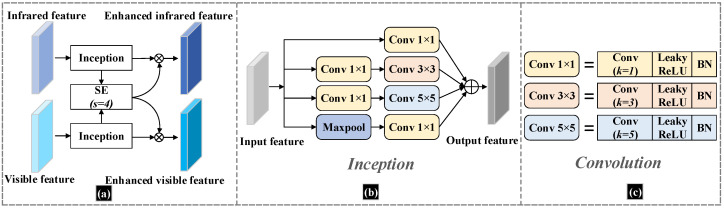
The structure of the Attention fusion module. (**a**) shows the data flow structure of the attention fusion. (**b**) shows the Inception structure in (**a**), which mainly connects the 4 branches. (**c**) shows the detailed description of the convolution structure in (**b**).

In the feature fusion module, we feed the visible and infrared images into a two-branch backbone and perform shared learning of features at the FPN layer. This architecture is used to achieve the fusion of mixed modal features of infrared and visible images. In the fusion module, we add the batch normalization (BN) operation to the double branch’s features to improve the network’s generalization ability. In addition, we add the SE attention module in the independent branches, which multiplies the attention feature vectors obtained from the two feature calculations with the corresponding branches. Moreover, we use the deep separable convolution instead of the conventional 3 × 3 convolution to reduce the number of parameters in the network with less network performance. The structure of the feature fusion module we designed to incorporate the attention mechanism is shown in Figure 4.

We can understand the attention fusion structure intuitively in Figure 4, where Figure 4a shows the main structure of the attention fusion module. The Attention fusion module is designed to enhance the information exchange between the infrared and visible channels as well as the mutual feature enhancement. The Inception module is designed to obtain multi-scale features in both infrared and visible images. It can also reduce the computational overhead while ensuring the accuracy of the network, thus improving the efficiency of the feature extraction network. In the structure shown in Figure 4a, we add the SE attention module to enhance the infrared and visible features. In this case, we set the squeeze factor of the SE module to s = 4. In particular, we designed the SE module by weighting the feature vectors of the infrared images with the features extracted from the visible image, resulting in attention feature maps for the visible image channels. Similarly, the attention feature maps for the infrared image channel are obtained by weighting the features with the feature vectors derived from the visible image channels by SE calculations. The structure of the Inceptive module in Figure 4a is shown in Figure 4b, and we use the Inception structure from [25]. The composition of the convolution part in Figure 4b is shown in Figure 4c. For each convolution operation, we use the Leaky ReLU activation function. Moreover, in the end, we add the BN operation. Enhanced feature maps, calculated by the attention fusion module, will be more favorable for later fusion.

#### 2.2.2. Fusion Shuffle Module

**Figure 5:**
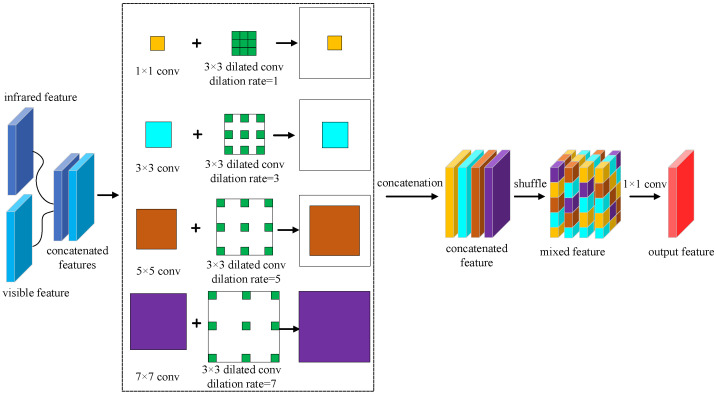
The fusion shuffle module structure where the shuffle is performed after fusion.

After the infrared features are fused with the visible features, we add the process of fusion shuffle. The purpose is to allow the network to learn more mixed features of the infrared and visible images, thus allowing the network to adapt to both modes of features. So we take the module’s design for feature enhancement from [26] and improve it. The Fusion shuffle module we designed is shown in Figure 5. As can be seen from the figure, after obtaining the infrared and visible features in the lower dimension, we concatenate the two features to create a double effect on the feature channel. We then add multiple branches of convolution layers with different kernel sizes and followed each convolution with a dilated convolution with the corresponding dilation rate. Finally, we concatenate the output of the four branches and then shuffle these enhanced features to form the mixed enhancement.

In Figure 5, we first design a four-branch convolution layer (including 1 × 1 convolution, 3 × 3 convolution, 5 × 5 convolution, and 7 × 7 convolution). Where 1 × 1 convolution and 3 × 3 convolution extract small object features in infrared images and visible images, 5 × 5 convolution extracts medium-scale object features, and 7 × 7 convolution aims to extract large-scale object features. The four-branch convolution structure enhances the depth features of the infrared and visible images. To further extend the field of perception for image feature extraction in both modes, we introduce additional dilated convolution in each branch. The aim of introducing dilated convolution is to generate feature maps with high resolution and make them more contextual. The intent is also to reduce computational costs. As the dilation rate setting of the dilated convolution, for the 1 × 1 convolution, we set the dilation rate of the 3 × 3 dilated convolution to 1. For the 3 × 3 convolution, we set up a 3 × 3 dilated convolution with a dilation rate of 3. For the 5 × 5 convolution, we set up a 3 × 3 dilated convolution with a dilation rate of 5. For the 7 × 7 convolution, we set up a 3 × 3 dilated convolution with a dilation rate of 7. The larger the dilation rate of the dilation convolution, the larger the perceptual field. Dilated convolutions with different dilation rates make the branches more focused on enhancing features of a particular size. After enhancing the features, we cascade four branches of features and performed a shuffle operation. Finally, we use a 1 × 1 convolution operation to reshape the output of the fused features.

### 2.3. Loss Function

In the design of the loss function, we divide the loss of Dual-YOLO into four parts. The first is for the D-fusion module. In the overall structure of the network, we design four fusion modules for visible and infrared images based on the feature pyramid structure. Furthermore, the corresponding fusion is carried out according to deep and shallow features. Assuming that the feature matrix of the visible image is $Z_{vis}$ and the feature matrix of the infrared image is $Z_{inf}$, the feature entropy of the two images $H_{i}\left(Z_{vis}\right)$ and $H_{i}\left(Z_{inf}\right)$ are calculated as shown in Equations (4) and (5).

(4) $$H_{i}\left(Z_{inf}\right)=C_{i}\left(Z_{vis},Z_{inf}\right)-D_{i}\left(Z_{vis}\|Z_{inf}\right)$$

(5) $$H_{i}\left(Z_{vis}\right)=C_{i}\left(Z_{inf},Z_{vis}\right)-D_{i}\left(Z_{inf}\|Z_{vis}\right)$$

where $C_{i}\left(Z_{vis},Z_{inf}\right)$ is the cross-entropy of the low-dimensional feature vectors $Z_{vis}$ and $Z_{inf}$ of the i-th D-fusion module. $D_{i}\left(Z_{inf}\|Z_{vis}\right)$ is the relative entropy of $Z_{vis}$ and $Z_{inf}$. In the loss of the D-fusion module, we add up the losses of the four different scales of the module and end up with a loss of $L_{fusion}$ the fusion module, as shown in Equation (6).

(6) $$\begin{array}{cc} L_{fusion}= & \sum_{i=1}^{4}\left(H_{i}\left(Z_{inf}\right)+H_{i}\left(Z_{vis}\right)\right)\\ = & \sum_{i=1}^{4}\left(C_{i}\left(Z_{vis},Z_{inf}\right)+C_{i}\left(Z_{inf},Z_{vis}\right)-D_{i}\left(Z_{vis}\|Z_{inf}\right)-D_{i}\left(Z_{inf}\|Z_{vis}\right)\right) \end{array}$$

The value of $L_{fusion}$ represents the number of pseudo-features in the visible image. By optimizing $L_{fusion}$, the parameters of the network for extracting features can be optimized. It is also possible to eliminate redundant image features, thus improving the network’s generalization ability and facilitating rapid convergence. For the coordinate position error, we choose Complete IoU (CIoU) Loss as the loss function, making the box-objective regression more stable, as shown in Equation (7).

(7) $$L_{box}=L_{CIoU}=1-IoU+\frac{\rho^{2}\left(b,b^{gt}\right)}{c^{2}}+\alpha \nu$$

where IoU is the intersection ratio of the prediction bounding box to the Ground True (GT) bounding box.

(8) $$IoU=|\frac{b\cap b^{gt}}{b\cup b^{gt}}|$$

(9) $$\nu =\frac{4}{\pi^{2}}{\left(\arctan\frac{w^{gt}}{h^{gt}}-\arctan\frac{w}{h}\right)}^{2}$$

where *b* is the predicted box, $b^{gt}$ is the GT box, ρ is the distance between the centroid of the predicted box and the GT box, *c* is the diagonal length of the smallest enclosing rectangle of the predicted box and the GT box, ρ is the similarity of the aspect ratio of the predicted box and the GT box, and α is the influence factor of ν.

For the object coordinate position error, we choose the Smooth Binary Cross Entropy (Smooth BCE) loss with logits function to increase numerical stability, which is calculated as shown in Equation (10).

(10) $$L_{obj}=-\frac{1}{n}\sum_{i}^{n}\left[y_{i}\cdot \log\left(\sigma \left(x_{i}\right)\right)+\left(1+y_{i}\right)\cdot \log\left(1-\sigma \left(x_{i}\right)\right)\right]$$

(11) $$\sigma \left(x_{i}\right)=\frac{1}{1+\exp(-x_{i})}$$

For the loss function of object classification, we choose Focal loss as the loss function, as shown in Equation (12).

(12) $$\begin{array}{cc} L_{cls}= & \sum_{i=1}^{S^{2}}\sum_{j=1}^{B}1_{i,j}^{obj}\sum_{c\in class}\left[-p_{i}\left(c\right)\log\left({\hat{p}}_{i}\left(c\right)\right)-\left(1-p_{i}\left(c\right)\right)\log\left(1-{\hat{p}}_{i}\left(c\right)\right)\right] \end{array}$$

where ${\hat{p}}_{i}\left(c\right)$ and $p_{i}\left(c\right)$ represent with predicted and true value probabilities, respectively. The number of input image cells is $S^{2}$. *B* is the number of bounding boxes predicted for each cell. The value of $1_{i,j}^{obj}$ is 1 or 0, that is whether there is a detection object in the *j*-th bounding box of the i-th cell. We use 1 if it exists, 0 otherwise. For the total loss function design, we add up the loss function of the head part with the loss of the D-fusion. The total loss value of the network is finally obtained, which is calculated as shown in Equation (13). Where λ is the correction factor of the fusion loss.

(13) $$L_{total}=\lambda L_{fusion}+L_{box}+L_{obj}+L_{cls}$$

## 3. Experiment and Analysis

To test the performance of the infrared image object detection models Dual-YOLO proposed in this paper, we use the public DroneVehicle, KAIST, and FLIR infrared pedestrian datasets.

### 3.1. Dataset Introduction

#### 3.1.1. DroneVehicle Dataset

The DroneVehicle dataset [24] is a large UAV aerial vehicle dataset for annotation, which is used for tasks such as vehicle detection and vehicle counting. The dataset images are taken in environments ranging from day to night and contain both infrared and visible images. The entire annotated dataset has 15,532 pairs (31,064 images) and 441,642 annotated instances. Moreover, it contains realistic environment occlusion and scale variation.

#### 3.1.2. KAIST Dataset

The KAIST dataset [11] is a multispectral detection dataset constructed by Hwang et al. in 2015 with the primary aim of addressing the lack of pedestrian detection data in nighttime environments. The dataset is divided into 12 subsets. Where set00∼set05 are training data (set00∼set02 are daytime scenes; set03∼set05 are nighttime scenes), and set06∼set11 are test data (set06∼set08 are daytime scenes; set09∼set11 are nighttime scenes). The image resolution sizes are 640 × 512, containing a total of 95,328 images, each containing both visible and infrared images. The KAIST dataset captures several regular traffic scenes, including campus, street, and countryside, during daytime and nighttime, respectively, and contains 103108 dense annotations.

#### 3.1.3. FLIR Dataset

The FLIR dataset [12] contains more than 10K pairs of 8-bit infrared images and 24-bit visible images, including people, vehicles, bicycles, and other objects in the daytime and nighttime scenes. The infrared images’ resolution is 640 × 512, while the corresponding resolution of visible images varies from 720 × 480 to 2048 × 1536. We resize each visible image to 640 × 512 in our experiments. The default FLIR training dataset is used as our training dataset, and 20 color-thermal pairs from the FLIR validation set are randomly selected as the testing dataset. The dataset information we used for training and testing is summarized in Table 1.

### 3.2. Implementation Details

We utilize the YOLOv7 network as the main framework. Each image is randomly horizontally flipped with a probability of 0.5 to increase the diversity. The whole network is optimized by stochastic gradient descent (SGD) optimizer for 300 epochs with a learning rate of 0.005 and a batch size of 16. Weight decay and momentum are set to 0.0001 and 0.9, respectively. We implement our codes with the PyTorch framework and conduct experiments on a workstation with two NVIDIA GTX3090 GPUs. We summarize the setting of experimental environment and parameter as shown in Table 2. The hyper-parameters of the dataset we used in this article is shown in Table 3. There are equal numbers of infrared and visible images, while using these datasets for network training and testing, we perform data cleaning operations.

### 3.3. Evaluation Metrics

Precision, Recall, and mean Average Precision (mAP) are used to evaluate the detection performance of different methods. In the experiments of this paper, we mainly use the values of precision and recall to measure the network’s performance, which are calculated as shown in Equations (14) and (15).

(14) $$precision=\frac{\mathrm{TP}}{\mathrm{TP}+\mathrm{FP}}$$

(15) $$recall=\frac{\mathrm{TP}}{\mathrm{TP}+\mathrm{FN}}$$

For example, in the FLIR dataset for detecting persons and cars, TP (True Positive) represents the number of cars (or persons) correctly recognized as cars (or persons). FP (False Positives) means the number of samples that identified non-car instances (or non-person instances) as cars (or persons), and FN (False Negatives) indicates the number of samples that identified cars (or persons) as non-car instances (or non-person instances).

Average Precision (AP) refers to the area value of the P-R curve surrounded by coordinates. The closer the AP value is to 1, the better the detection effect of the algorithm. The calculation process of AP can be summarized as follows:

(16) AP=∫P(R)dR

The mAP indicates each class’s average value of AP, which is used to measure the performance of multi-class object detection tasks fairly. Therefore, the mAP is also adopted to evaluate the detection accuracy in our experiments. The mAP measures the quality of bounding box predictions in the test set. Following [27], a prediction is considered a true positive if the IoU between the prediction and its nearest ground-truth annotation is more extensive than 0.5. The IoU is calculated as shown in Equation (8).

### 3.4. Analysis of Results

#### 3.4.1. Experiments on the DroneVehicle Remote Sensing Dataset

To verify the detection effectiveness of our proposed Dual-YOLO method on small infrared objects, we conduct a series of experiments on the DroneVehicle dataset. The experimental results are shown in Table 4. Based on our observations, the freight car and van classes are very similar in shape in the DroneVehicle dataset. Therefore, many popular detection methods incorporate these two classes into the other three classes when conducting experiments on the DroneVehicle dataset to eliminate the error caused by fine classification. However, we chose the complete the DroneVehicle dataset when experimenting. In addition, we compare the performance with the current popular object detection methods, and the performance comparison is shown in Table 4.

In Table 4, we divide the modality of the data into Visible and Infrared. Table 4 shows that when only visible data is used for training, popular networks such as RetinaNet and Mask R-CNN can achieve the highest accuracy of 47.9%. The algorithm that achieves the highest accuracy when training infrared data is YOLOv7. Therefore, we choose YOLOv7 as the basic framework for Dual-YOLO. The highest accuracy YOLOv7 can achieve is 66.7%. The Dual-YOLO algorithm proposed in this paper can reach 71.8% accuracy on the DroneVehicle dataset. It is worth noting that when we test the Dual-YOLO algorithm, the test set is the infrared image test set. Our proposed model also has the highest detection accuracy of 52.9% and 46.6% for the two categories of freight car and van that are difficult to detect. This result also shows that the Dual-YOLO design is very robust. Moreover, the detection of small objects also has strong performance.

#### 3.4.2. Experiments on the KAIST Pedestrian Dataset

To further verify the effectiveness and robustness of our proposed Dual-YOLO, we conduct experiments on the challenging KAIST dataset. After comparing with some popular methods, our experimental results are shown in Table 5. Here, we mainly compare with PearlGAN. PearlGAN’s design idea is similar to ours, which uses infrared and visible image fusion information. However, unlike the Dual-YOLO we proposed, PearlGAN does not integrate infrared and visible features in this design. Instead, the two information sources are constrained by the loss function. Therefore, we can also be seen from Table 5 that the method that we choose to use features for fusion before detection and add loss constraint has a better performance on the KAIST dataset.

**Figure 6:**
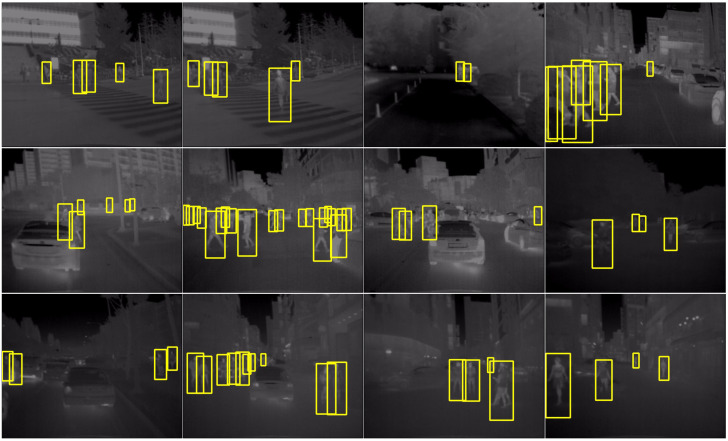
Visualization of Dual-YOLO detection results on the KAIST pedestrian dataset.

From Table 5, we can see that the accuracy of the other methods is low compared to the accuracy of our proposed network on the KAIST dataset. According to our analysis, this is due to the presence of many cluttered labels in the KAIST dataset, which leads to lower accuracy of other methods. However, we perform data cleaning on the dataset to remove pseudo-labels as well as incorrect labels before training the network. It can also be seen from the final results that our method also performs effectively in small infrared objects. Figure 6 shows the results of our tests and visualization of some data from the KAIST dataset. As can be seen from the figure, our network is highly robust to both changes in scale and changes in image brightness.

#### 3.4.3. Experiments on the FLIR Dataset

We also conduct a series of experiments on the FLIR dataset to prove the effectiveness of the proposed method. Furthermore, we compare the performance with some popular methods, such as SSD, RetinaNet, YOLOv5s, and YOLOF. The final experimental results are shown in Table 6.

Table 6 shows that our proposed method has the highest mAP value compared with other methods. The structure of Dual-YOLO we used is shuffled before fusion, which is also explained in detail in Section 3.5. From Table 6, we can also see that for small objects such as bicycles most methods have limited detection accuracy on such objects, such as SSD and RetinaNet. The Dual-YOLO we proposed has a strong detection effect for small and medium-sized objects such as persons. According to the data, the detection accuracy of our Dual-YOLO is 20.3% higher than that of YOLOv5s in the person category. We believe this improvement is not only due to the advancement of the YOLOv7 architecture. It shows that our idea of infrared and visible image fusion detection is reasonable. It is worth noting that the detection accuracy of the proposed network is up to 93.0% on the car class. Such detection accuracy is 13.0% higher than YOLOv5s and 7.5% higher than TermalNet in Table 6. According to our analysis, the visible image channel is added in our proposed Dual-YOLO so that the network can better recognize texture features. The enhancement of texture features makes the overall detection effect more optimized. It is worth mentioning that our proposed Dual-YOLO method increases by 4.5% compared with the YOLO-FIR on the overall mAP. YOLO-FIR is also designed based on the fusion of infrared and visible images. However, we design the attention fusion module and fusion shuffle module in the fusion process, which also increases our detection accuracy.

**Figure 7:**
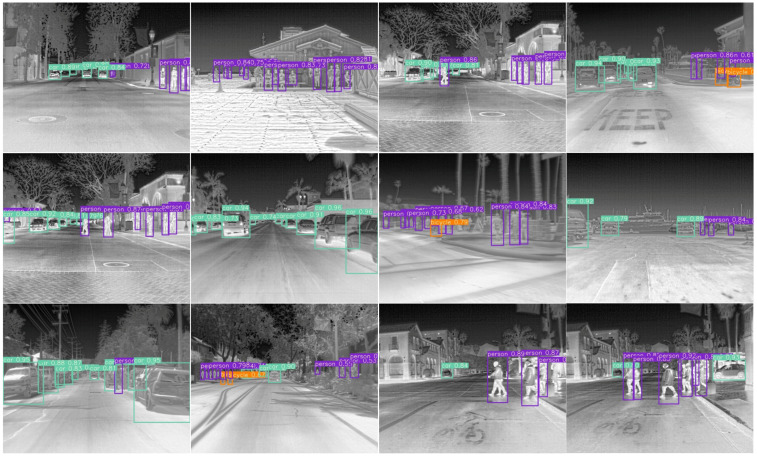
Visualization of Dual-YOLO detection results on the FLIR dataset.

Figure 7 shows some visualization results of the object detection effect on the FLIR dataset. From the fourth scene in the first row and the first scene in the third row in Figure 7, we can also see that for the objects with overlapping and occluded areas, our Dual-YOLO can fully detect cars. In the second scene in the second row, our detector can accurately detect overlapping objects and recognize objects with different scales. In this scenario, cars can be large or small, and our detector can detect them accurately. In the second scenario in the third row, our network also performs well in detecting small-sized objects such as bicycles. The surrounding scene in infrared images easily drowns the bicycle features. Therefore, it is challenging to detect this kind of object. Model complexity and runtime comparison of Dual-YOLO and the plain counterparts are shown in Table 7.

### 3.5. Ablation Study

#### 3.5.1. Position of the Shuffle

**Figure 8:**
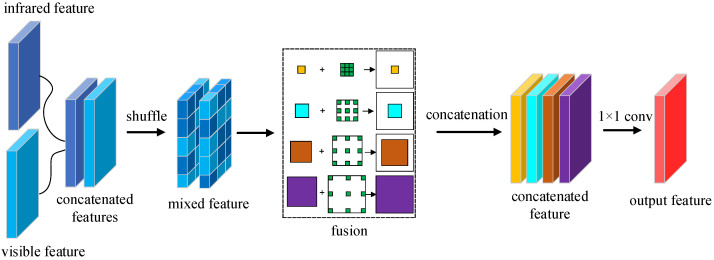
The fusion shuffle module structure where the shuffle is performed before fusion.

In the structure shown in Figure 5, we use the strategy of channel shuffle in the design of the fusion module. This strategy increases the exchange of feature information between different channels. Nevertheless, we have also considered whether shuffles should be used before or after fusion. As shown in Figure 8, we have placed the shuffle operation before the convolution fusion module to obtain a more blended feature. This processing is performed in such a way as to obtain information on the effective blending of the infrared and visible image before the convolutional fusion is performed. Therefore, we also conducted a set of experiments for validation. On the FLIR dataset, we carry out three different types of experiments.

The experimental results obtained according to the position of the shuffle are shown in Table 8. In the first row of Table 8 is the experiment without adding the shuffle fusion module, and the final obtained detection accuracy is 81.1%. The second row shows the experiments with the addition of the shuffle fusion and the placement of the shuffle operation after the convolutional fusion, resulting in an accuracy of 83.2%. Furthermore, the last line is where we added the shuffle fusion module and placed the shuffle operation before the convolutional fusion, resulting in an accuracy of 84.5%. Compared to the module without the addition of shuffle fusion, the accuracy of the network improved by 3.4% with the addition of this module. For the shuffle position, we can also conclude from Table 8 that there is a 1.3% improvement in the accuracy of the network when the shuffle operation is performed before the convolutional fusion.

#### 3.5.2. Functions of the Components in the Attention Fusion Module

**Figure 9:**
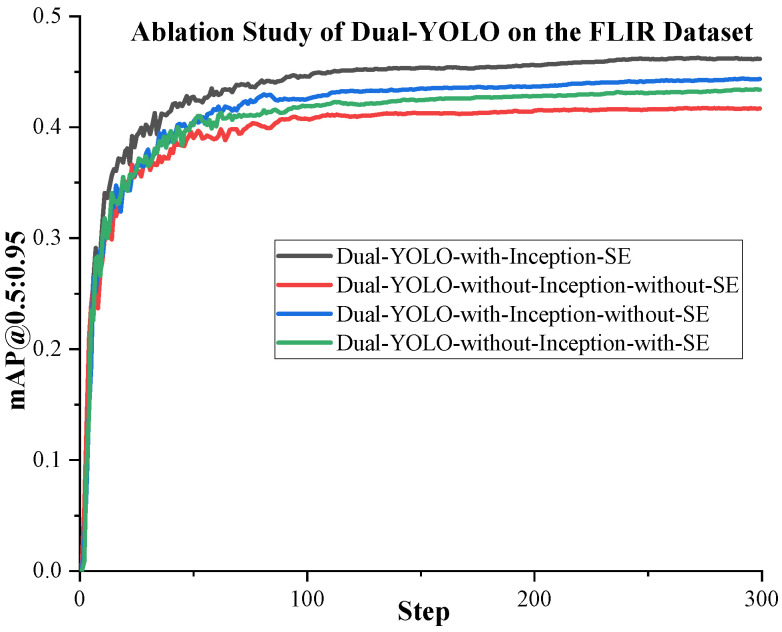
The mAP@.5:0.95 performance curve of Dual-YOLO during training. From the curves, we can see that Dual-YOLO has the highest accuracy when it adds Inception and the SE module together.

**Figure 10:**
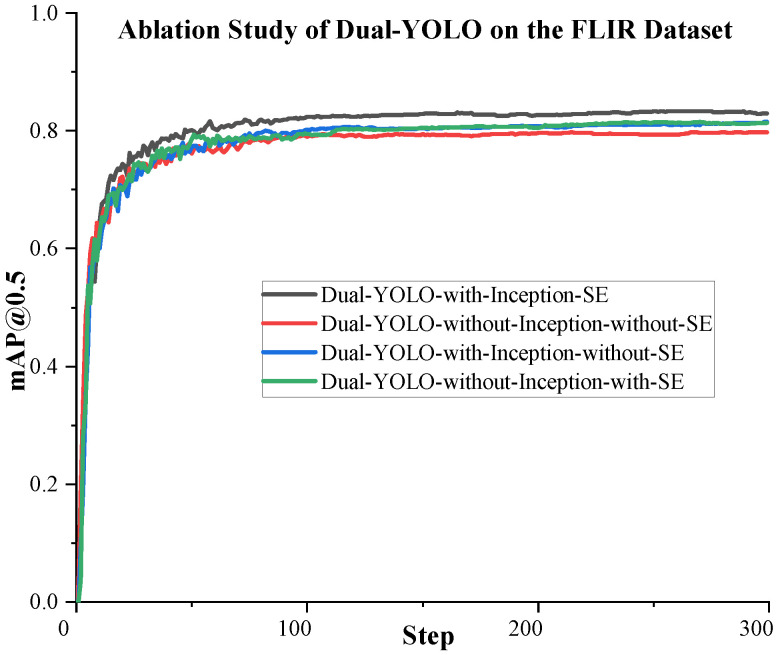
The mAP@0.5 performance curve of Dual-YOLO during training. From the curves, we can see that Dual-YOLO has the highest accuracy when it adds Inception and the SE module together.

We conduct the following ablation study to test the function of the attention fusion module proposed in Section 2.2 and its components. It is worth noting that there are four D-Fusion modules in our proposed Dual-YOLO network. In this ablation experiment, we perform the same configuration on the attention fusion module in each D-Fusion module. That is, the configuration of the four D-Fusions is precisely the same. Through experiments, the results obtained are shown in Table 9. The training curves of the proposed algorithms are shown in Figure 9 and Figure 10. In Table 9, we test the accuracy of Dual-YOLO on the FLIR dataset by adding or not adding Inception and SE modules. In order to eliminate the influence of different IoU Settings on the experiment, we not only used mAP@0.5 to evaluate the accuracy in this experiment but also used mAP@0.5:0.95 as the evaluation standard.

From Table 9, we can see that when we add Inception and SE modules in Dual-YOLO, the highest mAP is achieved on the FLIR dataset. After adding Inception and SE, mAP@0.5 is a 4.8% improvement over not adding these two modules. We can also see that the mAP@0.5 of the model increases by 1.4% when only SE is added. With Inception only, mAP@0.5 increased by 2.8%. We achieve the highest accuracy when we use Inception and SE modules together.

## 4. Conclusions

To overcome the problem of accuracy loss caused by the loss of texture features of infrared objects, we propose the Dual-YOLO object detection network with infrared and visible image fusion based on YOLOv7. In the infrared image feature extraction, we design the infrared and visible image feature fusion module named D-fusion. Furthermore, we obtain simplified and useful fusion information in feature extraction through attention fusion and fusion shuffle design. This method reduces the impact of redundant information on network accuracy reduction. Finally, we design the fusion module loss function in the network training process to accelerate the network’s convergence. Through experimental verification on the DroneVehicle, KAIST, and FLIR datasets, we prove the effectiveness of Dual-YOLO in improving the accuracy of infrared object detection. The proposed method is expected to be applied in the fields of military reconnaissance, unmanned driving, agricultural fruit detection, and public safety. Meanwhile, further research will include infrared and visible image fusion for semantic segmentation and infrared object tracking. In addition, we will do more optimization work in terms of parameter compression and acceleration of the model. Through these optimization strategies, the proposed infrared small target detection model Dual-YOLO is more suitable for embedded platform.

## Figures and Tables

**Table 1:** Dataset information we used in this paper.

Hyper-Parameter	DroneVehicle Dataset	KAIST Dataset	FLIR Dataset
Scenario	drone	pedestrian	adas
Modality	R + I	R + I	R + I
#Images	56,878	95,328	14,000
Categories	5	3	4
#Labels	190.6 K	103.1 K	14.5 K
Resolution	840 × 712	640 × 480	1600 × 1800
Year	2021	2015	2018

**Table 2:** Environment and parameter setting for the experiment setup.

Category	Parameter
CPU Intel	i9-10920X
GPU	RTX 3090 × 2
System	Ubuntu 18.04
Python	3.7
PyTorch	1.10
Training Epochs	300
Learning Rate	0.005
Weight Decay	0.0001
Momentum	0.9

**Table 3:** The hyper-parameters of the dataset we used in this manuscript. *test-val* means that the test set used in this article is the same as the validation set.

Hyper-Parameter	DroneVehicle Dataset	KAIST Dataset	FLIR Dataset
Visible Image Size	640 × 512	640 × 512	640 × 512
Infrared Image Size	640 × 512	640 × 512	640×512
#Visible Image	10,000	9853	10,228
#Infrared Image	10,000	9853	10,228
#Training set	9000	7601	8862
#Validation set	500	2252	1366
#Testing set	500	2252 (test-val)	1366 (test-val)

**Table 4:** Evaluation results on the DroneVehicle dataset. All values are in %.The top results are marked in green.

Method	Modality	Car	Freight Car	Truck	Bus	Van	mAP
RetinaNet(OBB) [28]	Visible	67.5	13.7	28.2	62.1	19.3	38.2
Faster R-CNN(OBB) [29]	Visible	67.9	26.3	38.6	67.0	23.2	44.6
Faster R-CNN(Dpool) [28]	Visible	68.2	26.4	38.7	69.1	26.4	45.8
Mask R-CNN [30]	Visible	68.5	26.8	39.8	66.8	25.4	45.5
Cascade Mask R-CNN [31]	Visible	68.0	27.3	44.7	69.3	29.8	47.8
RoITransformer [27]	Visible	68.1	29.1	44.2	70.6	27.6	47.9
YOLOv7 [3]	Visible	98.2	41.4	70.5	97.8	44.7	68.5
RetinaNet(OBB) [32]	Infrared	79.9	28.1	32.8	67.3	16.4	44.9
Faster R-CNN(OBB) [29]	Infrared	88.6	35.2	42.5	77.9	28.5	54.6
Faster R-CNN(Dpool) [28]	Infrared	88.9	36.8	47.9	78.3	32.8	56.9
Mask R-CNN [30]	Infrared	88.8	36.6	48.9	78.4	32.2	57.0
Cascade Mask R-CNN [31]	Infrared	81.0	39.0	47.2	79.3	33.0	55.9
RoITransformer [27]	Infrared	88.9	41.5	51.5	79.5	34.4	59.2
YOLOv7 [3]	Infrared	98.0	31.9	65.0	95.8	43.0	66.7
UA-CMDet [24]	Visible + Infrared	87.5	46.8	60.7	87.1	38.0	64.0
Dual-YOLO (Ours)	Visible + Infrared	98.1	52.9	65.7	95.8	46.6	71.8

**Table 5:** Pedestrian detection results of the synthesized images obtained by different translation methods on the KAIST dataset computed at a single IoU of 0.5. All values are in %. The top results are marked in green.

Method	Precision	Recall	mAP
CycleGAN [33]	4.7	2.8	1.1
UNIT [34]	26.7	14.5	11.0
MUNIT [35]	2.1	1.6	0.3
ToDayGAN [36]	11.4	14.9	5.0
UGATIT [37]	13.3	7.6	3.2
DRIT++ [38]	7.9	4.1	1.2
ForkGAN [39]	33.9	4.6	4.9
PearlGAN [21]	21.0	39.8	25.8
Dual-YOLO (Ours)	75.1	66.7	73.2

**Table 6:** Object detection results of the synthesized images obtained by different translation methods on FLIR dataset, were computed at a single IoU of 0.5. All values are in %. The top results are marked in green.

Method	Person	Bicycle	Car	mAP
Faster R-CNN [40]	39.6	54.7	67.6	53.9
SSD [1]	40.9	43.6	61.6	48.7
RetinaNet [32]	52.3	61.3	71.5	61.7
FCOS [4]	69.7	67.4	79.7	72.3
MMTOD-UNIT [40]	49.4	64.4	70.7	61.5
MMTOD-CG [40]	50.3	63.3	70.6	61.4
RefineDet [41]	77.2	57.2	84.5	72.9
TermalDet [42]	78.2	60.0	85.5	74.6
YOLO-FIR [9]	85.2	70.7	84.3	80.1
YOLOv3-tiny [16]	67.1	50.3	81.2	66.2
IARet [16]	77.2	48.7	85.8	70.7
CMPD [22]	69.6	59.8	78.1	69.3
PearlGAN [21]	54.0	23.0	75.5	50.8
Cascade R-CNN [31]	77.3	84.3	79.8	80.5
YOLOv5s [10]	68.3	67.1	80.0	71.8
YOLOF [43]	67.8	68.1	79.4	71.8
Dual-YOLO (Ours)	88.6	66.7	93.0	84.5

**Table 7:** Model complexity and runtime comparison of Dual-YOLO and the plain counterparts.

Method	Dataset	#Params	Runtime (fps)
Faster R-CNN (OBB)	DroneVehicle	58.3 M	5.3
Faster R-CNN (Dpool)	DroneVehicle	59.9 M	4.3
Mask R-CNN	DroneVehicle	242.0 M	13.5
RetinaNet	DroneVehicle	145.0 M	15.0
Cascade Mask R-CNN	DroneVehicle	368.0 M	9.8
RoITransformer	DroneVehicle	273.0 M	7.1
YOLOv7	DroneVehicle	72.1 M	161.0
SSD	FLIR	131.0 M	43.7
FCOS	FLIR	123.0 M	22.9
RefineDet	FLIR	128.0 M	24.1
YOLO-FIR	FLIR	7.1 M	83.3
YOLOv3-tiny	FLIR	17.0 M	50.0
Cascade R-CNN	FLIR	165.0 M	16.1
YOLOv5s	FLIR	14.0 M	41.0
YOLOF	FLIR	44.0 M	32.0
Dual-YOLO	DroneVehicle/FLIR	175.1 M	62.0

**Table 8:** On the FLIR dataset, object detection results at a single IoU of 0.50 when the shuffle is placed in different positions of Dual-YOLO. All values are in %.The top results are marked in green.

Method	Person	Bicycle	Car	mAP
without shuffle	87.2	63.6	92.6	81.1
shuffle before fusion	88.0	68.6	92.9	83.2
shuffle after fusion	88.6	66.7	93.0	84.5

**Table 9:** Object detection results of the synthesized images obtained by different modules in the attention fusion module on the FLIR dataset. These results are computed at a single IoU of 0.50 and IoU between 0.50 and 0.95. All values are in %.The top results are marked in green.

Inception	SE	Person	Bicycle	Car	mAP@0.5	mAP@0.5:0.95
✘	✘	85.1	64.5	89.4	79.7	41.6
✔	✘	86.9	69.0	91.6	82.5	44.3
✘	✔	86.2	65.7	91.4	81.1	43.3
✔	✔	88.6	66.7	93.0	84.5	46.2

## Data Availability

The DroneVehicle remote sensing dataset is obtained from https://github.com/VisDrone/DroneVehicle, accessed on 29 December 2021. The KAIST pedestrian dataset is obtained from https://github.com/SoonminHwang/rgbt-ped-detection/tree/master/data, accessed on 12 November 2021. The FLIR dataset is obtained from https://www.flir.com/oem/adas/adas-dataset-form/, accessed on 19 January 2022.
